# Rorschach Assessment in Suicide Survivors: Focus on Suicidal Ideation

**DOI:** 10.3389/fpubh.2018.00382

**Published:** 2019-01-11

**Authors:** Arianna Palmieri, Johann Roland Kleinbub, Stefania Mannarini, Sara Molinaro, Cristina Castriotta, Paolo Scocco

**Affiliations:** ^1^Department of Philosophy, Sociology, Education and Applied Psychology, University of Padova, Padova, Italy; ^2^Padova Neuroscience Center, University of Padova, Padova, Italy; ^3^Interdepartmental Center for Family Research, University of Padova, Padova, Italy; ^4^Soproxi Onlus, Padova, Italy; ^5^Mental Health Center, ULSS 6 Euganea, Padova, Italy

**Keywords:** suicide, survivor, Rorschach, suicidal ideation, health risk

## Abstract

**Background:** The study of Suicidal ideation (SI) in people bereaved through suicide (Suicide Survivors, SSs) could be hampered by the person's willingness to admit it, or by their limited awareness of it. Our main hypothesis is that SI is common in these people, especially if they are parents or children of the victim. For its potential in shedding light on specific unconscious processes, Rorschach test was chosen for our investigation, for the first time in SSs literature. Rorschach suicide ideation and selected variables were further analyzed to better delineate their psychological profile.

**Method:** Rorschach according to Exner's Comprehensive System was administered to 21 people bereaved through suicide presenting as outpatients at SOPROXI Project Service—Padova Mental Health Center- and 23 healthy controls. Beck Depression Inventory (BDI) was routinely administered to SSs and considered in the study.

**Results:** *T*-tests showed significantly higher mean SI score (S-Con) as it emerged from the Rorschach test S-Con scores in SSs compared to control participants. SI found only weak correlation with the BDI item in which SSs can explicitly state the desire for their death. Within-group analysis revealed higher S-Con mean scores in bereaved children and parents of the victim compared to other kind of kinships. Morbid content (MOR) has been fund as the most characterizing variable in SSs' S-Con in terms of effect size, followed by a low number of responses with an ordinary form (X +%). Human movements (M), Special Scores related to thought slippage (ALOG, FABCOM2, INCOM2, and CONTAM) and poor human representations (PHR) have been shown to be more significantly present in SSs compared controls.

**Discussion:** Psychodynamic interpretations of our results are provided. Clinical practice should consider Rorschach as one of eligible tools of investigation on this field.

## Introduction

A person who lost a friend, family member, or other loved ones through suicide have been also defined as suicide survivor (SSs). It has been described that a great percentage of SSs reported that the suicide of the one's beloved had significantly “disrupted” their lives (1).

According to an anecdotal figure often reported in literature spanning the last 30 years, about six people are left behind following every suicide. However, a recent study by Cerel et al. (2), on a huge U.S. sample, calculated that each suicide resulted in an average of 135 people exposed (i.e., who knew the deceased person) during their lifetime (resulting, in that case, in 5.5 million of people only in the Kentucky State).

This estimation provides an idea of the strong need of deepening the comprehension of the psychological consequences of being a person bereaved through suicide, in order to inform and guide the clinical practice.

A comprehensive review by Sveen and Walby (3) suggested that SSs report higher levels of rejection, shame, stigma, and blaming than other bereaved people, while sharing with them complicated grief, depression, PTSD symptoms, anxiety, and suicidal behavior. Among the psychological reactions to the loss of a beloved person, suicidal ideation (SI) has indeed been identified as one of the prominent features in SSs and it is still a challenging topic in this field. For instance, one of the first empirical contribution on SI in people bereaved through suicide, by Mitchell et al. (4), showed a strong association between complicated grief and SI, also controlling for depression levels.

More recently, in a large-sample study, Pitman et al. (5) found that SSs have a significantly increased risk of SI and suicide attempts compared with people bereaved by other sudden deaths. A possible issue in investigating the prominence of SI in this population is the fact that SI is generally hidden or neglected by a resistance to admit it or even acknowledging it (6–9).

In line with recent literature (4, 5, 10–12, 12–21), we hypothesize greater SI in people bereaved through suicide compared to a healthy control group. Specifically, we intend investigate SI through a projective test able to assess this clinical phenomenon even overcoming the participants' conscious or unconscious resistance to its expression, i.e., Rorschach Inkblot test coded and interpreted according to Exner's Comprehensive System (22, 23). We choose the Rorschach Inkblot test as it is based on the assumption that conscious and unconscious ways of feeling and thinking (namely cognitive, perceptual, affective, problem-solving, and coping resources) are reflected (projected) on the ambiguous materials of the test which can provide data about the person's functioning in the immediate present (24). According to a recent review by Kumar et al. (25), in the last 50 years the Rorschach test has been the most widely employed projective measure used to assess SI (22, 26, 27). Rorschach test has also indeed employed extensively in recent studies to characterize psychiatric populations and/or people suffering from seriously impacting bereavement [e.g., (28–33)], revealing psychological characteristics of patients who had escaped the research classically based on self-report questionnaires. For instance, Palmieri et al. (31) assessed with Rorschach test people affected by Amyotrophic Lateral Sclerosis, a terminal disease in which patients must face the anticipated mourning of themselves, highlighting greater SI than expected on the basis of self-report questionnaires or interviews (namely one third of the patients recruited in the sample showed high SI).

In detail, the CS is employed by 96% of health professionals using the Rorschach test (34), and contains a variables constellation specifically addressed to assess SI. Such constellation, named “S-Con,” demonstrated considerable predictive validity of self-destructive behaviors as well as near-lethal and lethal suicide behaviors (22, 26, 27, 35). Exner (22) reported that S-Con has been able to identify around 75% of suicidal patients (22). Hence. S-Con score was chosen as our main dependent variable. We expect only partial convergence between the patient's S-Con score and their explicitly expressed desire for death, considering interpersonal and intrapsychic resistance related to suicidal ideation.

To substantiate the hypothesized discrepancy between the SI and its explicitly expression, we used Beck Depression Inventory [BDI; (36)], routinely administered to these outpatients, by comparing their S-Con score and the score obtained at the BDI 9th item, which regards the idea of putting an end to one's own life. Our second hypothesis is that the expected greater SI in SSs participants, depends on the kinship with the deceased person, in terms of increasing SI if the victim is a child or a parent. The literature has already provided some elements to support the hypothesis that strict relatives bereaved by suicide may be at particular risk for suicide themselves (12, 13, 15, 18–21, 37).

Namely, it has been proved that a family history of suicide increases up to ten times the suicide risk in the family members (21) compared to the risk of general population, independently of possible inherited vulnerability for mental disorders shared with the victim (18). Recently, Campos et al. (13) and Santos et al. (19) found that, in a large European sample of SSs, being a strict relative of the suicided person, significantly contributed to the suicide risk. Similar results were found in Asian population: Song et al. (37) found that individuals who lost a family member have in their lifetime 4.5 times more probability of SI, whereas if the victim was a friend or an acquaintance the SI probability was, respectively 3.7 times and 2.2 times that of people without such experience.

Finally, to provide a more exhaustive picture, our third aim is to deepen into patients' Rorschach protocols. In the third part of the manuscript we present in detail all the S-CON variables scores to highlight which is the most characterizing SSs in such a constellation. In order to offer a profile of such population and to deeper investigate the way in which patients conceive others and relationships, we also investigated illogical combination of ideas (FABCOM2, INCOM2, CONTAM, ALOG), and poor human representations (PHR) as crucial variables to better delineate patients' ways of feelings and thinking, and total human movements responses (M), crucial, in our opinion for clinical practice in such at-risk population (details in Method section). Our expectation was that these variables were more frequent in patients than the control group. If significantly more present, these indexes would indicate a disturbance of thought, typical of patients who suffered a severe trauma and those at risk of suicide, aggression and dysphoric feelings in perceiving themselves and relationships with others, but also great resources in particular entering into empathic resonance with others, a typical aspect of those who survived from trauma suffering (38, 39). In detail, we introduced the latter variable, M, together with the other ones strongly aimed at highlighting psychopathological aspects, to investigate also the presence of a positive feature that could represent a basis for the reflection on the psychological interventions' planning in favor of these patients.

In synthesis, we mainly aimed to investigate the implicit suicidal ideation in SSs patients through the administration of a projective test, the Rorschach Inkblot Test based on CS.

Our first hypothesis was that the Suicide Constellation score should be higher in these patients compared to healthy controls. Furthermore, we compared patients S-CON's score to the score of one specific item of the BDI, expecting a lack of clear association between the explicit and projective-based assessment of SI.

Secondly, we hypothesized a higher S-CON score in the case of being a parent or a child of the suicide victim, compared to other kind of bonding.

Thirdly, to further delineate SSs profile, we deepened on S-Con variables to individuate which are the most characterizing this sample, and investigated further variables expected as peculiar in people bereaved through suicide.

To the best of our knowledge, this is the first study exploring SSs psychological features through a projective test: identifying, with such kind of assessment, the common features of people bereaved through suicide, mainly those related to SI, could be provided an original perspective helpful both for research and for clinical practice.

## Materials and Methods

### Participants

Between 2015 and 2017, eligible SSs were recruited consecutively from user of SOPROXI Project (40, 41). Soproxi project (www.soproxi.it) was established in Padua, Italy, in 2006, to offer information, support, treatment provision, awareness-building and educational campaigns, to people -mainly relatives and friends- who have experienced the suicide of someone close.

Inclusion criteria of our experimental sample were: be at least 18 years old, have lost someone close by suicide and be able to reach the outpatient office of Soproxi project at the Mental Health Center (Padova Hospital). Exclusion criteria were the presence of a frank cognitive impairment or other impediment to the understanding of the Italian language.

The Rorschach test according to CS procedure was administered to all 22 selected people according to inclusion and exclusion criteria. Since brief protocols (number of responses [R] < 14) are considered invalid, as Exner (42) reported that the temporal stability of CS scores was lower when the Rorschach protocols had fewer than 14 responses, one SSs' protocol was excluded from the study, resulting in 21 valid protocols.

Twenty-one control subjects were then recruited mainly from local voluntary associations. Same SSs inclusion and exclusion criteria were applied. No statistical differences were found between the two groups in terms of age, *t*_(39.37)_ = −0.39, *p* = 0.697, educational level, *t*_(38.47)_ = 0.15, *p* = 0.884, and gender distribution χ${}_{\left(1,\;\text{N}=42\right)}^{2}$ = 0, *p* > 0.999. All demographic data are reported in Table 1.

**Table 1:** demographics.

	**Patients**		**Controls**	
	**Mean**	***SD***	**Mean**	***SD***
Age (years)	46.05	12.48	47.67	14.18
Education (years)	12.9	4.61	12.71	3.77
Gender	F = 16		F = 15	
	M = 0 5		M = 0 6	

### Measures

#### Rorschach Test and Selected Variables

All Rorschach systems use the same set of 10 inkblot stimuli originally created by Rorschach (43), in which examinees are invited to look at each inkblot and say what it looks like or what it might be, giving one or more responses per inkblot. The Rorschach Comprehensive System [CS; (44)] we used in our study includes the CS suicide constellation “S-Con” (23), on which we focused our study, is an index composed by the number of variables exceeding (or being comprised within) specific threshold values, among a set of 12 variables (Sum VF + FD > 2; C-S Bl >0; Ego > 0.31>0,44; MOR > 3; Zd> ± 3,5; es>EA; CF + C > FC; X+% < 70; S < 3; *P* < 3 >8; Pure H < 2; R < 17); a value equal or > 8 in S-Con is considered as a cut-off sensitive in detecting people with significant suicidal ideations on the basis of empirical findings. Hence, it is suggested that the protocol of any person reaching the S-Con cut-off must be taken very seriously [for further details see (45–47)]. Namely, according to Mihura et al.' recent seminal review, the interpretation of each S-con variable can be synthetized as follows: high Vista and Dimension responses (Sum V + FD), which stand for depth perceived by gradations of dark and light or by shape, reflect negative self-perception, feelings of discomfort, jointly with personal introspection; high color over form responses (FC < CF+C) reflect self-control failure by an external or internal stimulation, placed by an exaggerated emotional reaction; High color shading blends (C-S Bl), which stands for color and its shading as seen simultaneously, reflects simultaneous experiencing joy and pain; Low Conventional Ordinary Form Quality (low X+%), which stands for low number of conventional perceptions of blot areas, reflects abnormal ability to perceive the world as others do, and maybe impaired reality testing; very high or very low Ego Index, which is a composite measure based on the number of reflections and pairs of objects, may be associated with an impaired ego organization and a capacity to meet internal and external demands and stressors: the Ego index can be either narcissistic or distress related (when high) or related to a negative self-image (when low); high white responses (S), provided when white background space is used in the response, reflects oppositional tendencies, and feelings of anger and aggression; High responses with damaged, dysphoric or morbid content (MOR) reflect morbid imagery and are associated with depressive and/or destructive thoughts or feelings; low or high Popular responses (P; i.e., the 13 most statistically common objects reported by at least one third of the normative sample) reflect, when low, unusual and, when high, stereotyped perceptions of reality; high or low scores in the processing efficiency (Zd), reflects, respectively excessive or impaired information processing or accounting; a low number of Images of whole, realistic human figures (Pure H), reflects an impaired perception of Self and others viewed as whole; Experienced Stimulation greater than Experience Actual (es > EA) reflects the current level of coping abilities in terms of experiencing more stress than what can be handled; finally a low number of total responses given to the whole protocol (R) reflects a limited ability to provide ideas, or solutions to a given issue.

Further four variables were considered in our analysis: the number of Human Movements (M), which reflect, respectively mental abilities (i.e., planning, empathic behaviors); moreover, since impaired reality testing and illogical combination of ideas can occur in loss and dysphoria, we grouped indices reflecting more serious forms of cognitive disarray (i.e., incongruous combination, INCOM level 2 + Fabulized combination, FABCOM level 2 + Contamination, CONTAM); finally, we considered poor Human Representation (poor H), i.e., Human or quasi-human images that are illogical, aggressive, damaged, or poorly formed, as they reflect disturbed and maladaptive understanding of others.

#### Beck Depression Inventory

The BDI (36) is one of the most widely used self-report inventory for measuring the severity of depression. It consists of 21 item on a 4-points Likert scale in which a score of 0 corresponds to “I don't have any thoughts of killing myself,” 1 corresponds to “I have thoughts of killing myself, but I would not carry them out,” 2 corresponds to “ I would like to kill myself” and 3 corresponds to “I would kill myself if I had the chance.”

### Procedure

The assessment took place in a clinical setting, and careful efforts were provided to make available for the participants emotional support during the interview. The Rorschach was administered by S.M (fourth author), a psychologist trained on Rorschach CS (third author of the study). The test administration lasted about 90 min.

Patients were given the choice to receive a brief report on the findings from their Rorschach protocol. Nine of them asked for it and received a written report. In 4 cases among these 9, patients requested and obtained a further clinical interview to discuss the reports content.

Since the BDI (36) was routinely administered to SSs attending at outpatient office of Soproxi project at the Mental Health Center, the item 9 of the inventory was employed as a measure of explicit expression of own death thoughts.

At the end of the whole process, five patients' protocols and five controls' ones were chosen at random and re-scored independently by the first author (A.P.), who was unaware of the fourth author's (S.M.) scores and was blind on the group belonging. The two sets of scored protocols were compared, and Kappa values were calculated for S-Con global index (Kappa = 0.94), M (Kappa = 1.0), and M- (Kappa = 1.0). Rater agreement on scoring the four signs was very high, demonstrating that these psychodynamic Rorschach variables can be reliably scored. Furthermore, for those who have overcome the clinical cut-off of suicidal ideation (namely, 3 people bereaved by suicide), team meetings have been held and, depending on the case, particular clinical attention has been dedicated to the theme of suicidal ideation in subsequent clinical interviews. Of note, all them had previously asked the Soproxi clinical team for a psychotherapy or clinical supportive aid. Informed consent to the aims of the study was obtained from each individual participant, in accordance with the guidelines of the 1995 Declaration of Helsinki [as revised in (48)]. This Soproxi Project has been approved by Ethics Committee of Clinical Experimental Projects of Padova Hospital (protocol number 0020095).

### Statistical Analyses

Cohen's Kappa was used to assess interrater reliability. One-tailed *t*-tests, using Welch approximation to the degrees of freedom for unequal variances, were performed to assess the difference in S-Con mean scores, respectively between patients and control participants and, between people bereaved through suicide that were parents or sons vs. other type of kinships with the victim. Further *t*-tests were performed to assess the difference between patients and controls in the M and PHR indexes, as well as a composite index obtained by the sum of Incom2, Fabcom2, and Contam indexes (IFC).

To further describe the difference between people bereaved through suicide and control groups, Cohen's d (49) for all S-Con components were reported to provide a standardized comparison scale. Extending the original classification of Cohen (49); Sawilowsky (50) provides the following classification of d values, which was employed in the analyses: d (0.01) = very small, d (0.2) = small, d (0.5) = medium, d (0.8) = large, d (1.2) = very large, and d (2.0) = huge.

## Results

Statistical analyses showed a significant difference, *t*_(39.69)_ = 2.45, *p* = 0.009, between the S-Con scores of SSs (*M* = 5.95, *SD* = 1.77) and control participants (*M* = 4.67, *SD* = 1.62), with patients having higher scores than controls, and a medium to large effect size (*d* = 0.76). Moreover, of 21 bereaved people who received the Rorschach test, 3 exceeded the cut-off rate of 8 score for the suicide ideation level, and 8 obtained a sub-threshold score between 5 and 7. In the control consisting of 23 subjects instead, instead no individual exceeded the cut-off, 5 subjects scored 5 and 2 scored 6.

The correlation between patients' BDI suicidal ideation item and their S-CON scores was found to be weak (*r* = 0.29). Moreover, the within patient's analysis showed a significant effect of kinship with the victim, *t*_(18.85)_ = 3.11, *p* = 0.003, with the sons or parents (*M* = 7.00, *SD* = 1.33) having a greater S-Con score than other type or relatives (*M* = 5.00, *SD* = 1.61), the Cohen's *d* = 1.35 indicates a very large effect.

“M” scores were found higher in SSs (*M* = 2.6, *SD* = 2.27) than in control participants (*M* = 0.79, *SD* = 0.85), *t*_(25.54)_ = 3.43, *p* = 0.001, with a large effect size (*d* = 1.06); PHR scores showed a similar result, *t*_(29.31)_ = 3.45, *p* < 0.001, with patients (*M* = 4.95, *SD* = 3.57) showing higher scores than controls (*M* = 1.95, *SD* = 1.77) and a large effect size (*d* = 1.06). Finally, the IFC composite index results showed patients (*M* = 0.81, *SD* = 1.12) presenting a positive mean score, while all control participants scored zero, *t*_(20)_ = 3.3, *p* = 0.002, *d* = 1.02.

Descriptive statistics and effect sizes of all S-Con components are reported in Table 2.

**Table 2:** Scores of S-Con components by group.

	**Patients**		**Controls**			
	**Mean**	***SD***	**Mean**	***SD***	**Cohen's d**	
sumVFD	1	0.95	1.55	1.59	0.42	Small
ColShdBl	8.24	4.68	6.48	4.64	0.38	Small
EgoIndex	0.42	0.24	0.35	0.16	0.33	Small
MOR	3.86	2.39	0.62	0.97	1.77	Very large
Zd	−1.55	5.78	−2.93	3.21	0.3	Small
esEA	5.62	6.19	4.07	6.92	0.24	Small
CFpCmFC	0.46	1.86	0.05	2.03	0.21	Small
X+%	41.24	13.69	52.62	14.88	0.8	Large
S	5.38	2.94	3.33	2.44	0.76	Medium
P	4.33	1.49	5.14	1.11	0.62	Medium
PureH	2.57	1.66	2.24	1.26	0.23	Small
R	26.62	8.63	23.38	7.53	0.4	Small

Ten patients scored “0” on the selected BDI item, other ten patients reported a score of “1,” and there was one response “2” and no response “3.” The correlation between this measure and S-Con scores was low, *r* = 0.22.

## Discussion

In line with our main hypothesis, we found a greater SI in patients bereaved through suicide when compared to control participants as assessed with Rorschach test according Exner's System, consistently with recent literature findings on the topic of SI in SSs (4, 5, 10–21, 37).

Of note, the presence of elevated suicidal ideation does not correspond *sensu strictu* to the consequent manifest suicidal act. It has been indeed documented the existence of a continuum in severity in suicidal ideation and behavior, ranging from death thoughts to suicide planning, with behaviors (attempted and completed suicide) being less frequent phenomena, that is the majority of people do not act on their suicidal ideation (7, 9, 51, 52). In this vein, Viglione and Hilsenroth (35), suggest the S-Con should not be used to rule out suicide risk but to increase awareness about self-destructive behavior and suicide. Both false-positives and false-negatives are indeed possible during objective assessments. From a health professional's point of view false positive in the domain of suicide assessment is not very disturbing but any false-negative case is a matter of grave concern. A false-negative case on objective assessment implicates that the individual has suicidal ideations but does not express it explicitly. In such situations, the use of projective tests becomes more important as on the ambiguous stimuli of these tests, the individual may indicate his/her suicidal ideations.

Anyhow, the high presence of this type of ideation in the SSs group also confirmed with the investigation based on a projective test confirms the intensity of this type of ideation and the need to further deepen this issue in people bereaved thorough suicide. The mental processes that are activated in the production of Rorschach responses, indeed, tap into and trigger the underlying personality structures of the respondent, which are not captured by self-report measures or interviews (53) because self-reports depend on the respondents' willingness to reveal the asked-for information about themselves, their perceived risk by revealing certain information, such as SI, and their self-knowledge.

We also found a lack of clear association between the score obtained with the S-Con and what was explicitly stated at item 9 of the BDI related to the desire for one's own death.

This fact corroborates the idea that IS in SSs may be outside of awareness, or there may be a strong resistance to admit it. According to scientific literature on the topic of suicide as a whole, thirty-seven of the patients attempting suicide had communicated their suicidal intentions to people around them, but all of them used only protracted indirect verbal communication (9). About one-third to one-half of all suicide victims have communicated their intent to family members, and a roughly similar proportion to health care professionals during the final few months (7). Furthermore, research has shown that the majority of high school students would tell a friend if they were thinking of suicide, not a parent or counselor (6, 8). In general perspective, it has been shown that there is a profound discrepancy between conscious and unconscious level regarding one's own mental states and the concept of health (54–56), especially in clinical subjects (57). This could indeed lead to underestimate the magnitude of the phenomenon, which could be even more pervasive than commonly thought.

Moreover, consistently with our second hypothesis, i.e., that within our group of people bereaved through suicide, being a parent or child of a suicide victim generates more SI than other types of relationship / kinship also according to Rorschach test results, the association with S-Con Score was significantly higher in the case of being a parent or a child of the victim.

Scientific literature has already provided evidence in this direction (12, 13, 15, 18–21, 37), noting associations between levels of psychopathology and degree of kinship with the victim of suicide. Even in these cases, however, the survey was based on self-report measures or by means of interviews based on explicit questions, and never before with the use of a projective measure.

The psychodynamic perspective can provide a meaningful interpretation of this second result, i.e., that being a parent or child of a suicide victim is associated to higher SI than other types of relationship / kinship. In the classical psychodynamic interpretation (58) the concept of secondary identification to which we refer in the following, typically occurs in mournful experiences: it allows the lost object (suicidal person, in this case) to survive in the ego of the one who remains. In this vein, the dynamic of identification in the parent-child relationship is intensely present and reciprocal in the parent/child transgenerational axis, and characterize, at different level, various stages of evolutionary development (59). In “Mourning and Melancholia,” Freud (58) theorized that the loss of a loved object generates an unconscious identification with that object, ambivalently perceived both desirable (i.e., “good”) than abandoning (i.e., “bad”). The outcome of this pathological identification is an unconscious confusion between the self and the lost object. In that process, some aspects of the ego are split-off and come to represent the abandoning object. Hence, aggressive feelings toward that lost, abandoning object are directed against the split-off aspect of the ego which serves as a stand-in for the object. These hostile feelings directed against the self can results in self-destructive fantasies. In the case of death by suicide, this dynamic can be particularly intense because, unlike other casual death or loss, the suicide victim—the lost object- appears to be a person who has consciously and voluntarily chosen to abandon his/her loved child or parent. In a further psychodynamic interpretation (60, 61), the introjected aggression toward the original love object is enlisted by the Super-ego, fueling attacks against the ego. In this formulation the Super-ego, in which arises sense of guilt and desire of auto-punishment, acts a relentless attack on the ego. In this case as well, the psychic dynamics can be exacerbated in the people bereaved through suicide, in which the sense of inadequacy and guilt for not having done enough to save the suicide victim from the choice of the extreme act can be enormous in the case of a children or a parent. Asch's words (62) elegantly sum up this perspective: “much of the meaning of the usual suicidal act can be understood once we recognize that there is frequently a double aim of first cleansing the self, and then uniting (actually reuniting) with an omnipotent love object” (p. 52).

In line with this psychodynamic premise, it follows that suicidal ideation can be more activated when the suicide victim is a child or a parent. In the first case, it is perhaps superfluous to say that surviving one's own children represents a huge conflict, and it is common clinical experience that is perceived by the parents as being “against nature.” Even more, when the child voluntarily chooses to end his life, this act can be perceived by the parents as an existential failure. The literature has in fact been very dedicated to this theme, highlighting the particular drama of this condition (12, 13, 15, 18–21, 37). In the second case, relating to the death by suicide of a parent, the drama and the anger facing the self can be similarly enormous, because the SS was not able to save the life to who gave life to him/her.

As a third objective of our study we were focused in investigating the S-Con variables resulting to have the widest effect size. The greatest one is the morbid content (MOR), revealing as the most characterizing variable among those constituting S-Con in our SSs sample. MOR is a code used for any response in which an object is identified as dead, destroyed, damaged, injured, or characterized by clearly dysphoric feeling (63). As MOR responses pertains most directly to issues of self-image, its incisive presence signals that patients' thinking is marked by a pessimistic set, thus implicating a tendency to conceptualize the self and the relationship to the world with a sense of discouragement. Exner (63) highlight that when MOR is particularly high in a protocol, pessimism can be joint to disorganized ideation. Interestingly, as Since S-Con has been validated only in adult population, Silberg and Armstrong (27) searched for an experimental index designed to detect SI in adolescents, founding that MOR, alone, discriminates suicidality in adolescents as well. In trauma research literature, particularly rich in empirical studies based on evaluation with the Rorschach test, MOR content variables have been indeed often associated with traumatic experiences (64) and it has been included in the “Trauma Index” (65). Our result is therefore in line with the pathognomonic relevance that the MOR index has also revealed in other contexts of study.

The second variable revealing a remarkable effect size among those of S-Con is Conventional Ordinary Form Quality (X+%), with mean SSs' mean scores lower than those of controls. The X+% represent the proportion of formal ordinary responses in the protocol. When its score is within normal range, it means that the person mediational decisions tend to be common or conventional. When it is low, as in this case, it signifies that the person tends to translate stimulus field in atypical ways. Such a prominence in SSs can be usefully interpreted jointly to the other selected indices besides S-Con. In detail, the sum of indices reflecting severe cognitive disarray (INCOM level 2 + FABCOM level 2 + CONTAM). As a whole, the occurrence of these marked in SSs variables can reflect an impaired reality testing and thought disorder, similarly to what has been often found in trauma research based on Rorschach assessment (66, 67). Ephraim (68) highlight, in this vein, that cognitive disturbances are associated with intrusive recollections, and underlined the importance of acknowledging their trauma-related nature, seen as important indications of the potential perceptual idiosyncrasies and difficulties with reality testing of traumatized individuals, as in the case of SSs.

A higher number of poor Human representations (PHR), namely human or quasi-human images that are illogical, aggressive, damaged, or poorly formed were found, as expected, in our SSs Sample compared to controls. In this vain, Varvin and Rosenbaum (69) argued that the experience of helplessness and object loss are the two most salient aspects of psychological trauma. This concerns both the loss of important objects in the external world and the loss of what is conceptualized as internal representations of comforting objects.

This aspect is particularly fitting in the psychic dynamics that typically can involve a suicide survivor, as described in detail above.

Finally, as hypothesize, Human Movements (M) were found as higher in SSs compared to control subjects. M is mostly interpreted as an indication of resources, commonly related to how people view themselves and others and to empathy: an association between the presence of M responses and the activation of the mirror neural mechanism was indeed found (32), corroborating such evidence. As stated by Weiner (70), it might be the single component of the test most revealing of the individual's role in interpersonal relationships. Therefore, the high presence of M responses could suggest the presence of a marked sensitivity of SSs, typical of people who understand the pain of others because they have experienced direct trauma and loss: such attitude has been highlighted both in clinical and in neuroscientific perspective (38, 39).

In summary, as regards the third purpose of this study, the variables among those that seem to best characterize the constellation of suicidal ideation are the responses with morbid content (MOR), which reflect an image of oneself and of the damaged and dysphoric world, and the low number of ordinary form content, reflecting a distort, or at least unconventional, individuals' perception of the world. Consistently with the analyses of the other selected variables, marked illogical thinking, higher when compared to that of control group, indicating “traumatic thought disorders” emerged in our SSs sample. It can be seen as important indications of the potential perceptual idiosyncrasies and difficulties with reality testing by SSs, jointly to a distorted perception of the self and the others, probably characterized by anger.

The significant presence of these indices, as a whole, is well suited to the characteristics already described in these SSs, as previously emerged from self-report measures studies, such as depression, complicated grief and PTSD symptoms, other than suicidal ideation [e.g., see (3), for a review]. In these psychopathological frameworks, apparently distinct, some clinical signs and symptoms overlaps in terms of negative thoughts about yourself, other people or the world, self-destructive behavior, irritability, angry outbursts, or aggressive behavior, and distorted perception of reality caused by numbness or detachment.

The indices provided by our Rorschach study could hence represent, in future research, the starting point for identifying a composite index or a pathognomonic constellation useful to systematically detect the main psychopathological cues of suffering in SSs.

Of note, positive signs of marked empathic abilities and high cognitive resources were also found, as expected, in our sample. In this vein, Cerel and colleagues yet underlined SSs' motivation to help others like themselves (71), highlighting the fact that support groups are the most frequently utilized form of treatment for most people bereaved by beloved one's suicide. However, with a few exceptions, the investigation of the SSs' positive psychological resources is almost always placed in the background in research, which is mainly aimed at investigating their psychopathological reactions. It should be also considered that scientific literature is very fruitful in the study of psychological prevention and postvention of SSs [for a review see (3)], but many of the studies aimed at testing the effectiveness of the interventions are inspired by the main patients' psychopathological cues to outline targeted interventions, as in the case, for instance, by Testoni et al. (72), who suggest to focus on forgiveness toward the facilitation of the elaboration of self-blame.

Acting with awareness, non-judging and non-reacting seem to be dimension with a protective effect on psychological distress in SSs; for this reason, Mindfulness-based weekend retreats could be a further effective intervention in alleviating the suffering of this particular population of users (73).

To improve the patient's condition focusing indeed on the pathological dimensions and on the source of suffering is undoubtedly the main road on planning efficacious psychological intervention; however, to implement clinical approaches also considering the individuals' peculiar resources could represent a strength point in an effective intervention planning strategy. In this case, the SSs' ability to empathize and the cognitive complexity wealth inherited from their suffering, as evidenced by the high number of M responses, could be a potential starting point the intervention strategies.

In conclusion, one of the main messages that this article intends to provide is to not underestimate the SI in the SSs, even when this was explicitly denied by them, especially if the victim of suicide is a parent or a child. Rorschach test scored and interpreted according CS (22), can represent a useful tool in such a psychodiagnostic investigation.

As recently emerged in the study by Pitman et al. (5), indeed, the physical health of SSs is poor, and odds mortality ratio are higher to those reported in the normal population. In this context, one of the future research directions could be aimed at better investigating the causes of the mortality of SSs. It is not possible, in fact, to exclude in some situations the presence of “masked suicides,” or that people unconsciously risk their physical safety without incurring a deliberate suicidal act, according the unconscious psychodynamics described above, that would lead to a reunion with the loved object putting at the same time an end to the pain and supporting self-punitive drives (58, 60, 62). Among the many implications on health care which arise from our argumentations, the hospital staff and its setting, in particular the psychiatric one, to which patients bereaved by suicide often address to, has a pivotal role. As stated by Jordan and McMenamy (74), the psychiatric hospital setting is one of the elective setting for diagnosis and for the planning of treatments -both at the psychotherapy and pharmacotherapy level- for this population which, in turn, is at risk of suicide.

Our study suffers from some limitations, such as the reduced number of subjects recruited in our clinical sample and the lack of an additional control group, in addition to that constituted by healthy subjects. A third representative group of a bereaved people for sudden loss of loved ones in a different way from suicidal act would have allowed a comparison useful to better delineate the specific characteristics of SSs' sample. Further studies are warranted to fill the questions that these limits can inherently raise. A further limitation of the study is to have investigated a population of bereaved by suicide who asked for a psychological support, therefore only partially representative of the entire population of the SSs.

Despite these limitations, our first study using the Rorschach test in the people bereaved by suicide is hoped to have contribute to better delineate some psychological characteristics of these individuals, and in particular to focus the attention of health professionals toward the need to actively assess and be vigilant mainly in terms of suicidal ideation in at-risk population such as suicide survivors.

## Data Availability Statement

The raw data supporting the conclusions of this manuscript will be made available by the authors, without undue reservation, to any qualified researcher.

## Author Contributions

AP and PS designed the study and interpreted the results. PS, SaM and CC carried out the study and acquitted the data (with the direction of PS). JRK and StM conceived the statistical design and performed the data analyses. AP wrote the main part of the article. All authors helped to shape the research, critically revised the article and gave their final approval.

### Conflict of Interest Statement

The authors declare that the research was conducted in the absence of any commercial or financial relationships that could be construed as a potential conflict of interest.
